# Experimental Assessment of the Time-Dependent Protective Effects of Ivermectin Against Atezolizumab-Induced Ovarian Toxicity and Impaired Reproductive Function

**DOI:** 10.3390/toxics14100876

**Published:** 2026-09-30

**Authors:** Sukran Kozali Uzun, Seval Bulut, Bahadir Suleyman, Renad Mammadov, Taha Abdulkadir Coban, Mustafa Ozkaraca, Halis Suleyman

**Affiliations:** 1 Department of Obstetrics and Gynecology, Faculty of Medicine, Erzincan Binali Yıldırım University, Erzincan 24100, Turkey; sukran.uzun@erzincan.edu.tr; 2 Department of Medical Pharmacology, Faculty of Medicine, Erzincan Binali Yıldırım University, Erzincan 24100, Turkey; 3 Department of Biochemistry, Faculty of Medicine, Erzincan Binali Yıldırım University, Erzincan 24100, Turkey; 4 Department of Pathology, Faculty of Veterinary Medicine, Sivas Cumhuriyet University, Sivas 58140, Turkey

**Keywords:** atezolizumab, ivermectin, ovarian toxicity, oxidative stress, inflammation, anti-Müllerian hormone, ovarian injury

## Abstract

Atezolizumab, an immune checkpoint inhibitor, may induce oxidative stress, inflammation, and tissue injury in addition to its antitumor effects. This study investigated the protective effects of ivermectin against atezolizumab-associated ovarian toxicity and their dependence on treatment duration. Four experimental groups comprising six female Wistar albino rats were established: healthy (HG), atezolizumab (ATZ), ivermectin plus atezolizumab for 3 days (IVAZ-3), and ivermectin plus atezolizumab for 6 days (IVAZ-6). Ivermectin was administered orally at 2 mg/kg/day, followed one hour after the final dose by atezolizumab (10 mg/kg, intraperitoneally). Three days after atezolizumab exposure, animals were euthanized with high-dose ketamine. Serum prolactin and anti-Müllerian hormone (AMH) were measured, while ovarian tissues were analyzed for malondialdehyde (MDA), total glutathione (tGSH), superoxide dismutase (SOD), catalase (CAT), tumor necrosis factor-alpha (TNF-α), and interleukin-1 beta (IL-1β), along with histopathological and immunohistochemical changes. Compared with HG, ATZ increased MDA, TNF-α, and IL-1β and decreased tGSH, SOD, and CAT, with severe ovarian degeneration and increased caspase-3, inducible nitric oxide synthase (iNOS), IL-1β, and microtubule-associated protein-1 light chain 3-beta (LC3B) immunopositivity. IVAZ-3 significantly attenuated these alterations and preserved AMH, whereas most parameters in IVAZ-6 remained comparable to ATZ. Thus, ivermectin exhibited a treatment-duration-dependent protective effect, most pronounced with the 3-day regimen.

## 1. Introduction

Atezolizumab is an immune checkpoint inhibitor (ICI) that enhances T-cell responses by blocking programmed death-ligand 1 (PD-L1) and is widely used in the treatment of various malignancies [1,2,3]. Despite their survival benefits, the clinical application of these agents may be constrained by the development of immune-related adverse events [4]. Atezolizumab has been associated with toxicities affecting various organ systems, including the cardiovascular, gastrointestinal, and hepatic systems, and may also exert adverse effects on the reproductive system [5,6]. Although uncommon, cases of ovarian failure have been reported in association with immune-related endocrine toxicities [7]. It has been suggested that the PD-1/PD-L1 signaling pathway plays a role not only in tumor immune evasion but also in the regulation of immune homeostasis within reproductive tissues, including the ovary [8]. Experimental studies have demonstrated that PD-1 blockade can induce increased T-lymphocyte infiltration and upregulation of cyclooxygenase-2 (COX-2) and tumor necrosis factor-alpha (TNF-α) expression in ovarian tissue, and that these inflammatory alterations may contribute to follicular loss and the development of ovarian injury [9,10]. Consequently, inhibition of this immune checkpoint may disrupt the physiological immune balance within ovarian tissue, thereby promoting inflammatory responses and contributing to impaired ovarian function [10]. Furthermore, atezolizumab has been reported to increase intracellular calcium (Ca^2+^) and reactive oxygen species (ROS) levels while reducing adenosine triphosphate (ATP) concentrations, and hypercalcemia has also been observed in some patients receiving this treatment [11,12,13]. It is recognized that Ca^2+^, ATP, and oxidative stress are interrelated, with these processes capable of mutually amplifying one another and thereby exacerbating cellular damage [14]. On the other hand, the PD-1/PD-L1 signaling pathway has been demonstrated to play a role in regulating mitochondrial metabolism, oxidative balance, and cellular energy homeostasis, whereas its blockade may alter mitochondrial function and ROS production [15,16]. A recent study also reported that atezolizumab may adversely affect oxidative stress and antioxidant defense systems, while ATP supplementation markedly attenuated the oxidative and histopathological changes [17]. Taken together, these findings suggest that the toxic effects of atezolizumab on the reproductive system may be associated with the interplay among inflammation, oxidative stress, alterations in Ca^2+^ homeostasis, and dysregulation of mitochondrial energy metabolism. Accordingly, antioxidant-based and anti-inflammatory strategies may represent a promising approach for mitigating such tissue damage.

Avermectins are antiparasitic agents derived from Streptomyces species and are known for their activity against a broad range of arthropods and nematodes. Ivermectin, a semisynthetic derivative of avermectins, is widely used for the treatment of parasitic infections in humans and domestic animals [18]. The antiparasitic activity of avermectins is primarily mediated through their binding to glutamate-gated chloride channels in invertebrates, resulting in neuronal hyperpolarization that ultimately leads to paralysis and death of the parasite [19]. At low concentrations, these compounds may also modulate ion channels associated with gamma-aminobutyric acid, glycine, and nicotinic acetylcholine receptors [20,21]. Recent experimental evidence suggests that ivermectin protects reproductive tissues by enhancing antioxidant defenses, improving hormonal balance, modulating apoptotic processes, and attenuating histopathological injury in the ovaries and uterus [22]. Furthermore, ivermectin has been shown to attenuate lipopolysaccharide-induced increases in TNF-α, nitric oxide, prostaglandin E_2_ release, and intracellular Ca^2+^ levels [23]. Notably, it has been reported to suppress the expression of inflammation-related mediators, including iNOS and COX-2 [24]. In addition, ivermectin has been shown to enhance mitochondrial ATP production through the induction of Cox6a2, a subunit of the respiratory chain [25]. Collectively, these findings suggest that ivermectin may help maintain cellular ATP homeostasis and thereby support the preservation of physiological cellular functions. However, it has also been reported that the effects of ivermectin on mitochondrial function may vary depending on the dose and duration of exposure. Prolonged or high-concentration exposure to ivermectin has been shown to be associated with reductions in cellular ATP levels, a decrease in mitochondrial membrane potential, and an increase in ROS production [26,27]. Taken together, these apparently contrasting findings raise the possibility that the protective effect of ivermectin against the potential ovarian toxicity of atezolizumab may vary depending on the timing of its administration, making treatment duration a potentially critical determinant of efficacy.

In this context, ivermectin was hypothesized to exert a protective effect against ovarian injury induced by atezolizumab by attenuating inflammatory activation and Ca^2+^ accumulation while supporting mitochondrial energy metabolism under appropriate conditions. Conversely, it was anticipated that short-term ivermectin treatment would mitigate atezolizumab-associated inflammatory and oxidative ovarian damage, whereas prolonged treatment might diminish or abolish this protective response. Therefore, the present study aimed to investigate the effects of ivermectin on atezolizumab-induced potential ovarian injury and reproductive dysfunction in rats and to determine whether its protective efficacy varies according to the duration of administration.

## 2. Materials and Methods

### 2.1. Animals

Female Wistar albino rats (*n* = 24; 10–12 weeks of age; 270–280 g) were used as the experimental subjects. The animals were supplied by the Experimental Animals Application and Research Center of Erzincan Binali Yıldırım University and housed under controlled laboratory conditions. Room temperature was maintained at approximately 22 °C, and the lighting schedule consisted of alternating 12-h periods of light and darkness. The 24 rats were randomly allocated to four experimental groups using a simple random allocation method, with six animals in each group (*n* = 6). Sample size was determined using the resource equation approach described by Arifin and Zahiruddin [28]. Before the initiation of the experimental procedures, all animals were allowed to acclimatize to the laboratory conditions for 7 days. The animals were provided standard laboratory chow manufactured by Bayramoğlu Yem San. ve Tic. Ltd. Şti. (Erzurum, Türkiye) and had free access to food and water throughout the study. All experimental procedures complied with the principles of European Directive 2010/63/EU concerning the use of animals for scientific purposes. In addition, the study methodology and reporting were structured according to the ARRIVE (Animal Research: Reporting of In Vivo Experiments) guidelines, thereby promoting methodological rigor and transparent reporting [29]. The study protocol was reviewed and approved by the Local Animal Experimentation Ethics Committee of EBYU (date: 25 June 2026).

### 2.2. Chemicals

The atezolizumab used in the present study was obtained in its commercially available formulation, Tecentriq (F. Hoffmann-La Roche Ltd., Basel, Switzerland; 1200 mg/20 mL), from the Oncology Clinic of EBYU Mengucek Gazi Training and Research Hospital. Ivermectin tablets (3 mg) were supplied by Humanis Sağlık A.Ş. (Tekirdağ, Turkey), while ketamine was obtained from Pfizer İlaçları Ltd. Şti. (Istanbul, Turkey).

### 2.3. Experimental Animals

The animals were allocated into four experimental groups: healthy group (HG), atezolizumab alone (ATZ), ivermectin plus atezolizumab with a 3-day treatment protocol (IVAZ-3), and ivermectin plus atezolizumab with a 6-day treatment protocol (IVAZ-6).

### 2.4. Experimental Procedures

For administration, 3-mg ivermectin tablets were finely pulverized and suspended in 5.5 mL of distilled water immediately before use. The resulting suspension (approximately 0.55 mg/mL) was administered by oral gavage at approximately 1 mL/rat, corresponding to 2 mg/kg. The ivermectin dose of 2 mg/kg/day administered orally was selected based on previous experimental studies reporting biological and protective effects at this dose in rodents [30]. The ivermectin treatment durations were determined based on previously reported experimental protocols and evidence of duration-dependent effects in rodents [31,32]. For the IVAZ-3 group, ivermectin (2 mg/kg) was given orally by gavage once daily for three successive days. One hour after the final ivermectin dose, the animals received atezolizumab at 10 mg/kg via intraperitoneal (i.p.) injection [17,33]. The IVAZ-6 gr received ivermectin orally by gavage at the same dose (2 mg/kg) once daily for six consecutive days. One hour after the final ivermectin dose on day 6, the IVAZ-6 animals received a single intraperitoneal injection of atezolizumab at 10 mg/kg. The ATZ group received atezolizumab alone as a single intraperitoneal dose of 10 mg/kg on day 6, without any ivermectin pretreatment. Thus, atezolizumab was administered as a single dose on day 6 in the ATZ, IVAZ-3, and IVAZ-6 groups, whereas the duration of ivermectin pretreatment differed between the IVAZ-3 and IVAZ-6 groups. Three days following atezolizumab exposure, all animals were euthanized with high-dose ketamine (120 mg/kg), and the ovarian tissues were harvested. The experimental schedule was arranged to ensure that atezolizumab treatment was performed on the same day in the IVAZ-3 and IVAZ-6 groups. Accordingly, ivermectin treatment in the IVAZ-6 group was initiated three days before that in the IVAZ-3 group, allowing atezolizumab to be given to both groups one hour after their respective final ivermectin doses. Prior to euthanasia, blood samples were collected from the tail veins, and serum prolactin and anti-Müllerian hormone (AMH) concentrations were subsequently determined. The harvested ovarian tissues were subsequently analyzed for malondialdehyde (MDA), total glutathione (tGSH), superoxide dismutase (SOD), catalase (CAT), TNF-α, and interleukin-1β (IL-1β). In addition, the tissues underwent histopathological and immunohistochemical assessment.

### 2.5. Biochemical Analyses

#### 2.5.1. Tissue Sample Preparation

After euthanasia, the ovaries were excised bilaterally from each animal. The ovaries were allocated according to the planned analyses, with the right ovary designated for biochemical measurements and the left ovary processed for histopathological and immunofluorescence evaluations. For biochemical assessment, the right ovary was immediately snap-frozen in liquid nitrogen and homogenized directly, with no intermediate storage or freeze–thaw cycle. Tissue homogenization was performed in phosphate-buffered saline (PBS, pH 7.4) using a 1:10 tissue-to-buffer ratio (*w*/*v*), followed by vigorous vortex mixing. The homogenates were subsequently centrifuged at 10,000× *g* for 15 min at 4 °C. After centrifugation, the supernatant was carefully separated, divided into aliquots, and maintained at −80 °C until further biochemical measurements.

#### 2.5.2. Determination of MDA, tGSH, SOD, CAT, and Protein Levels

Ovarian tissue levels of MDA, tGSH, SOD, and CAT were analyzed using rat-specific commercial enzyme-linked immunosorbent assay (ELISA) kits. MDA levels were quantified using the YL Biotech ELISA kit (Cat. No. YLA0029RA), while tGSH levels were measured with the corresponding kit (Cat. No. YLA0121RA). SOD and CAT levels were assessed using ELISA kits with Cat. Nos. YLA0115RA and YLA0123RA, respectively. All assays were conducted in accordance with the manufacturer’s instructions (Shanghai YL Biotech Co., Ltd., Shanghai, China). Briefly, tissue homogenate samples and standards were added to the appropriate wells and incubated according to the manufacturer’s instructions. Following the specified incubation period, the wells were washed, and the corresponding detection reagents were added. After the final incubation and substrate reaction, absorbance was measured using a microplate reader at the wavelength specified for each assay. Analyte concentrations were calculated from the respective standard curves and normalized to the total protein content of the corresponding tissue sample. Total protein concentration in the ovarian tissue homogenates was determined using the Bradford protein assay according to the method described previously [34]. Protein content was quantified prior to normalization of the biochemical findings. For this purpose, tissue homogenate samples were subjected to the Bradford protein assay, and the absorbance values obtained by spectrophotometry were converted to protein concentrations using a calibration curve prepared from the relevant protein standard. The resulting concentrations were subsequently used as the reference basis for expressing the levels or activities of the measured oxidative stress parameters, including MDA (nmol/mg protein), tGSH (ng/mg protein), SOD (ng/mg protein), and CAT (ng/mg protein).

#### 2.5.3. Determination of Inflammatory Marker Levels

TNF-α (ng/mg protein) and IL-1β (pg/mg protein) concentrations in ovarian tissue were assessed with commercially available ELISA kits specifically designed for rats. The YL Biotech kit (Cat. No. YLA0118RA) was used for TNF-α determination, while IL-1β was analyzed with the corresponding YL Biotech kit (Cat. No. YLA0030RA). The assays were performed in accordance with the protocols provided with the respective kits.

#### 2.5.4. Serum Prolactin and AMH Determination

Serum prolactin concentrations were quantified with an ARCHITECT immunoassay analyzer and the ARCHITECT Prolactin assay kit (Cat. No. 7K7625; Abbott Laboratories, Abbott Park, IL, USA).

Serum AMH concentrations were measured using the UNICELL-AMH assay kit (YHLO Biotech, Shenzhen, China), which is based on a fluorescence-based immunochromatographic sandwich assay. All analyses followed the manufacturer’s recommended procedures.

### 2.6. Immunohistochemical Analysis

For immunohistochemical staining, tissue sections of 5 μm thickness were transferred onto poly-L-lysine-treated glass slides. Paraffin was removed with xylene, after which the sections were passed through decreasing concentrations of ethanol to restore hydration. The slides were then rinsed in phosphate-buffered saline (PBS), and endogenous peroxidase was quenched by exposure to 3% H_2_O_2_ for 10 min. To facilitate antibody binding, heat-induced antigen unmasking was carried out with the appropriate retrieval solution at 500 W, using two successive 5-min heating cycles. After another PBS rinse, the sections were kept at 4 °C overnight in the presence of the primary anti-Caspase-3 antibody (Thermo Fisher Scientific, Cat. No. PA5-114687, Waltham, MA, USA), anti-iNOS (Affbiotech, Cat. No. AF0199, Abbott Park, IL, USA), anti-IL-1β (Santa Cruz Biotechnology, Cat. No. sc-52012, Dallas, TX, USA), and anti-LC3B (Santa Cruz Biotechnology, Cat. No. sc-271625), each applied at a dilution of 1:200. Visualization of the primary antibody-bound complexes was performed with the Large Volume Detection System: Anti-Polyvalent, HRP (Thermo Fisher Scientific; Cat. No. TP-125-HL), following the protocol specified for the detection reagent. Color development was achieved with 3,3′-diaminobenzidine (DAB). Following chromogenic development, the tissue sections were counterstained with Mayer’s hematoxylin and subsequently subjected to dehydration and clearing before mounting and permanently mounted with Entellan. Immunohistochemical staining was evaluated using light microscopy. The intensity and extent of immunopositivity were semi-quantitatively scored on a five-grade scale: 0, no staining; 1, mild; 2, moderate; 3, severe; and 4, very severe immunoreactivity.

### 2.7. Histopathological Analysis

Following euthanasia, the rats were subjected to necropsy, and the left ovarian tissues were excised and immediately fixed in 10% neutral buffered formalin. Following fixation, the ovarian specimens underwent conventional tissue processing involving sequential dehydration and xylene treatment before being embedded in paraffin wax. Paraffin blocks were then sectioned at a thickness of 5 μm, and the resulting sections were placed on poly-L-lysine-coated glass slides. Tissue morphology was subsequently evaluated after staining with hematoxylin and eosin (H&E) and examined microscopically. Histopathological changes, namely hyperemia, follicular degeneration, and parenchymal degeneration, were semi-quantitatively assessed in six microscopic fields selected at random. The severity of the observed lesions was graded using a four-point scoring system as follows: 0, absent; 1, mild; 2, moderate; and 3, severe.

### 2.8. Statistical Analysis

Statistical analyses were carried out with IBM SPSS Statistics version 27.0 (IBM Corp., Armonk, NY, USA). Biochemical data were summarized as means ± standard deviations (SD). Prior to group comparisons, the distribution of the data was assessed by the Shapiro–Wilk test, while Levene’s test was applied to examine the homogeneity of variances. The biochemical outcomes were evaluated by one-way ANOVA. Depending on the variance characteristics of each variable, different post hoc procedures were selected. Tukey’s test was applied to MDA, tGSH, SOD, and TNF-α, for which the variance criterion was fulfilled, whereas CAT and IL-1β were examined with the Games–Howell test because their variances were not comparable across groups. Histopathological and immunohistochemical findings were presented as median (25th–75th percentile) values because these data were ordinal in nature. Between-group differences were evaluated nonparametrically using the Kruskal–Wallis test. Where an overall difference was identified, pairwise analyses were subsequently conducted with the Mann–Whitney U test. A two-tailed *p*-value < 0.05 was considered indicative of statistical significance.

## 3. Results

### 3.1. Biochemical Results

#### 3.1.1. MDA Levels in Ovarian Tissues

**Figure 1:**
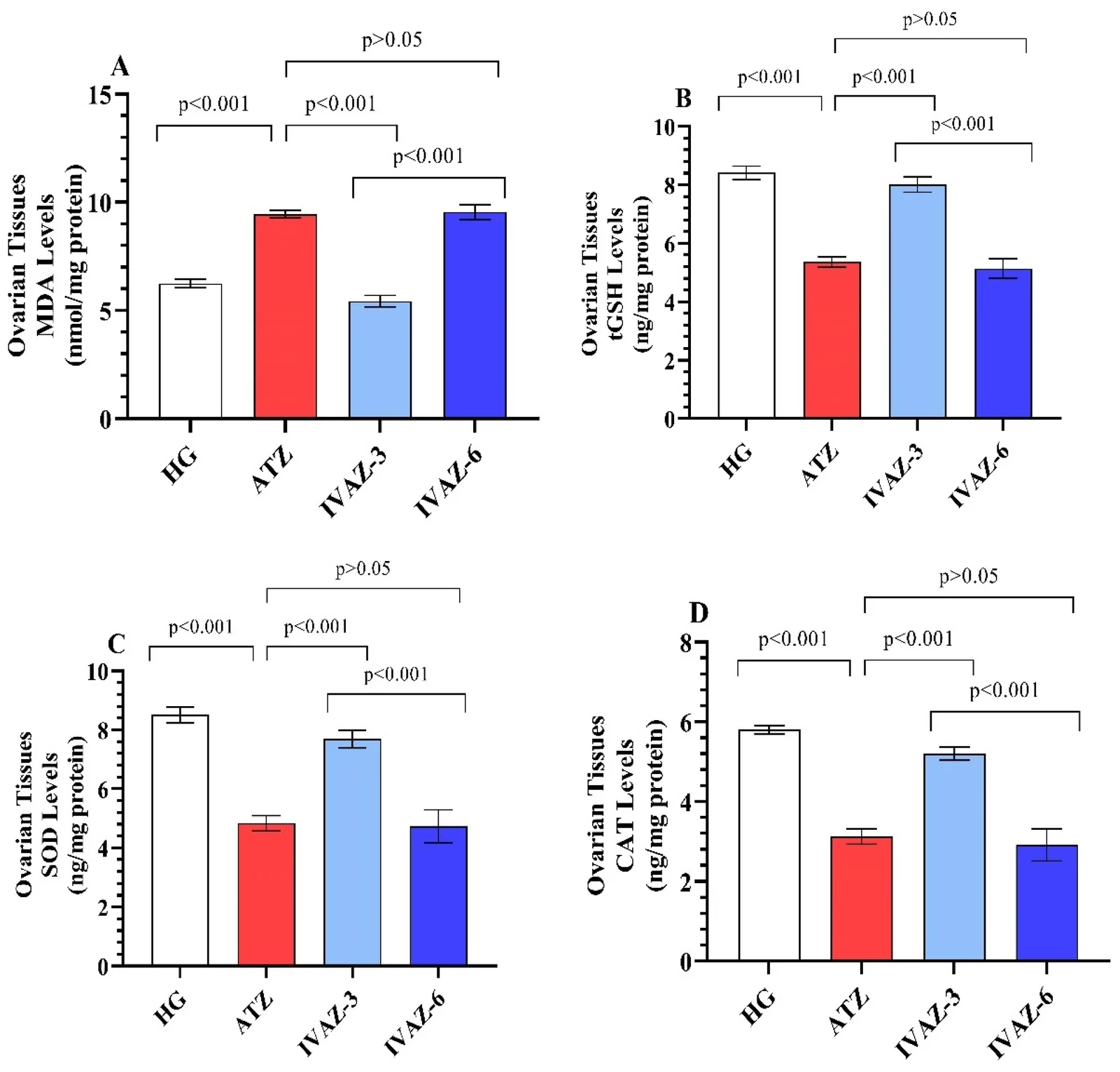
(**A**–**D**). Time-dependent effects of ivermectin treatment on MDA (**A**), tGSH (**B**), SOD (**C**), and CAT (**D**) levels in ovarian tissue of rats exposed to atezolizumab and treated with ivermectin for 3 or 6 days. Data are presented as means ± standard deviations (SDs), with *n* = 6 animals per group. MDA: malondialdehyde, tGSH: total glutathione, SOD: superoxide dismutase, CAT: catalase, HG: healthy group, ATZ: atezolizumab group, IVAZ-3: ivermectin + atezolizumab (3-day), IVAZ-6: ivermectin + atezolizumab (6-day). Statistical comparisons were conducted using one-way analysis of variance (ANOVA), followed by the appropriate post hoc test (Tukey or Games–Howell tests); *p* < 0.05 was considered statistically significant.

As evidenced in Figure 1A and Table 1, MDA levels were substantially higher in the ATZ group compared to the HG control (*p* < 0.001). This increase was successfully mitigated following short-term ivermectin administration, as evidenced by the IVAZ-3 group exhibiting lower MDA levels than the ATZ group (*p* < 0.01). However, extending the treatment to six days (IVAZ-6) failed to produce a comparable effect, showing non-significant differences relative to the ATZ group (*p* > 0.05). Overall, the findings suggest that the therapeutic impact on MDA attenuation is limited to brief exposure periods rather than prolonged treatment.

#### 3.1.2. tGSH Levels in Ovarian Tissues

As detailed in Figure 1B and Table 1, ATZ exposure led to a severe depletion of ovarian tGSH contents relative to the HG control (*p* < 0.001). Short-term ivermectin intervention effectively counteracted this decline; tGSH levels in the IVAZ-3 were markedly higher than in the ATZ group (*p* < 0.001) and restored to levels statistically indistinguishable from the HG group (*p* > 0.05). In contrast, extending the administration to six days (IVAZ-6) failed to produce a noticeable change compared to the ATZ group (*p* > 0.05). In summary, these observations imply that ivermectin’s protective effect on ovarian tGSH pools is restricted to brief treatment durations and dissipates with prolonged exposure.

#### 3.1.3. SOD and CAT Levels in Ovarian Tissues

The data compiled in Figure 1C,D and Table 1 highlight a marked suppression of both SOD and CAT activities in the ATZ cohort relative to the HG group (*p* < 0.001). A 3-day ivermectin regimen effectively counteracted this enzymatic dysfunction, with the IVAZ-3 group exhibiting significantly higher SOD and CAT levels than the ATZ control (*p* < 0.01). Conversely, prolonged administration in the IVAZ-6 group yielded no statistically meaningful deviation from the ATZ (*p* > 0.05). Altogether, these outcome metrics reveal that restoring antioxidant enzyme functionality depends on a brief treatment duration, whereas extending the protocol negates this beneficial response.

#### 3.1.4. TNF-α and IL-1β Levels in Ovarian Tissues

**Figure 2:**
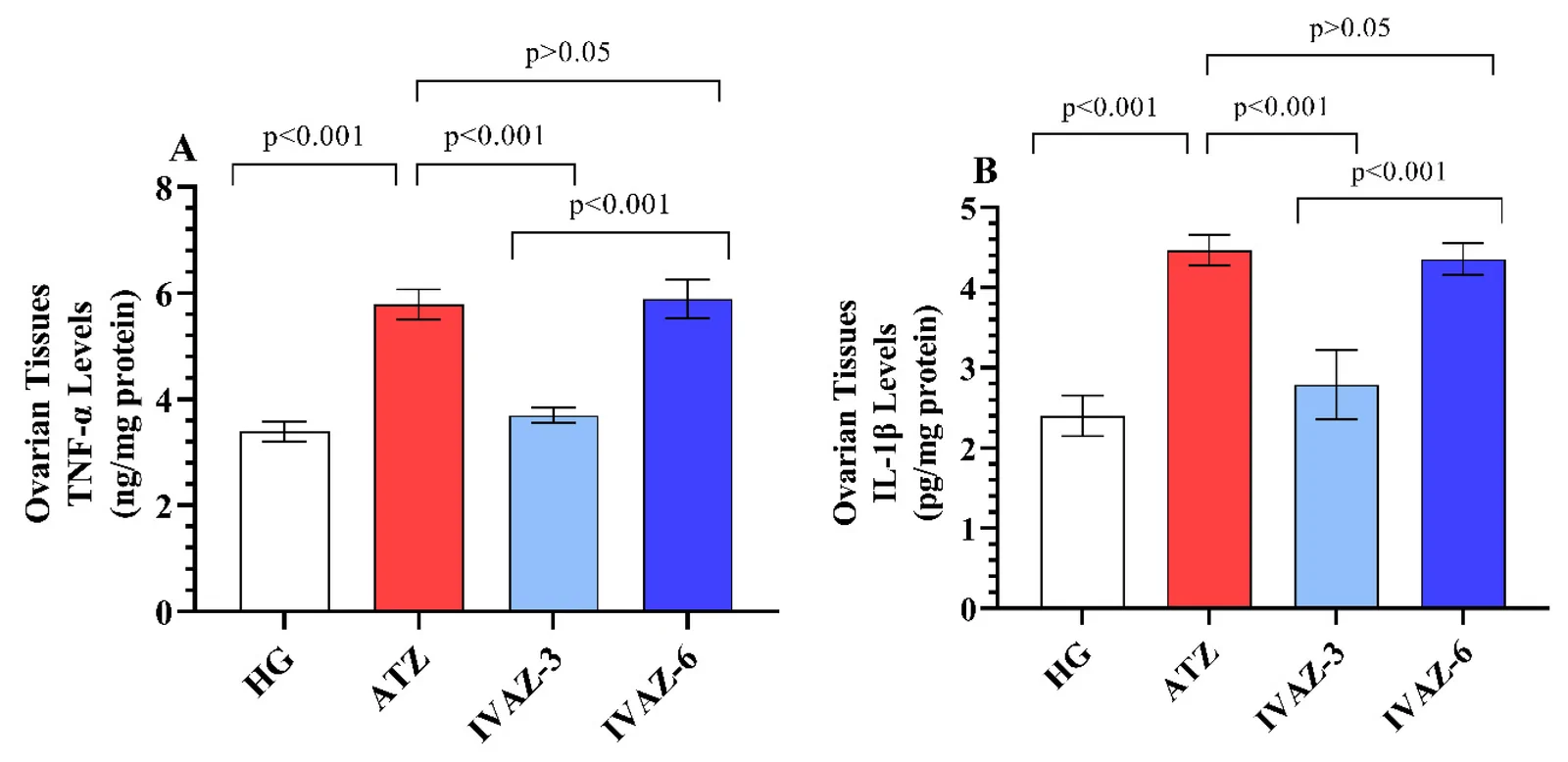
(**A**,**B**). Time-dependent effects of ivermectin treatment on TNF-α (**A**) and IL-1β (**B**) levels in ovarian tissue of rats exposed to atezolizumab and treated with ivermectin for 3 or 6 days. Data are presented as means ± standard deviations (SDs), with *n* = 6 animals per group. TNF-α: tumor necrosis factor-alpha, IL-1β: interleukin-1 beta, HG: healthy group, ATZ: atezolizumab group, IVAZ-3: ivermectin + atezolizumab (3-day), IVAZ-6: ivermectin + atezolizumab (6-day). Statistical comparisons were conducted using one-way analysis of variance (ANOVA), followed by the appropriate post hoc test (Tukey or Games–Howell tests); *p* < 0.05 was considered statistically significant.

Figure 2A,B and Table 1 demonstrate that ATZ exposure triggered a severe elevation in ovarian TNF-α and IL-1β concentrations compared to the HG control (*p* < 0.001). Short-term ivermectin therapy successfully suppressed this proinflammatory response, as the IVAZ-3 group displayed significantly reduced levels of both cytokines relative to the ATZ group (*p* < 0.001). Remarkably, TNF-α levels in the IVAZ-3 cohort were statistically indistinguishable from those in the HG group (*p* > 0.05). Conversely, extending ivermectin treatment to six days (IVAZ-6) yielded no significant deviation in either cytokine compared to the ATZ (*p* > 0.05). In summary, these data confirm that the anti-inflammatory efficacy of ivermectin is restricted to a shorter treatment window and disappears with extended dosing.

#### 3.1.5. Serum Prolactin and AMH Levels

**Figure 3:**
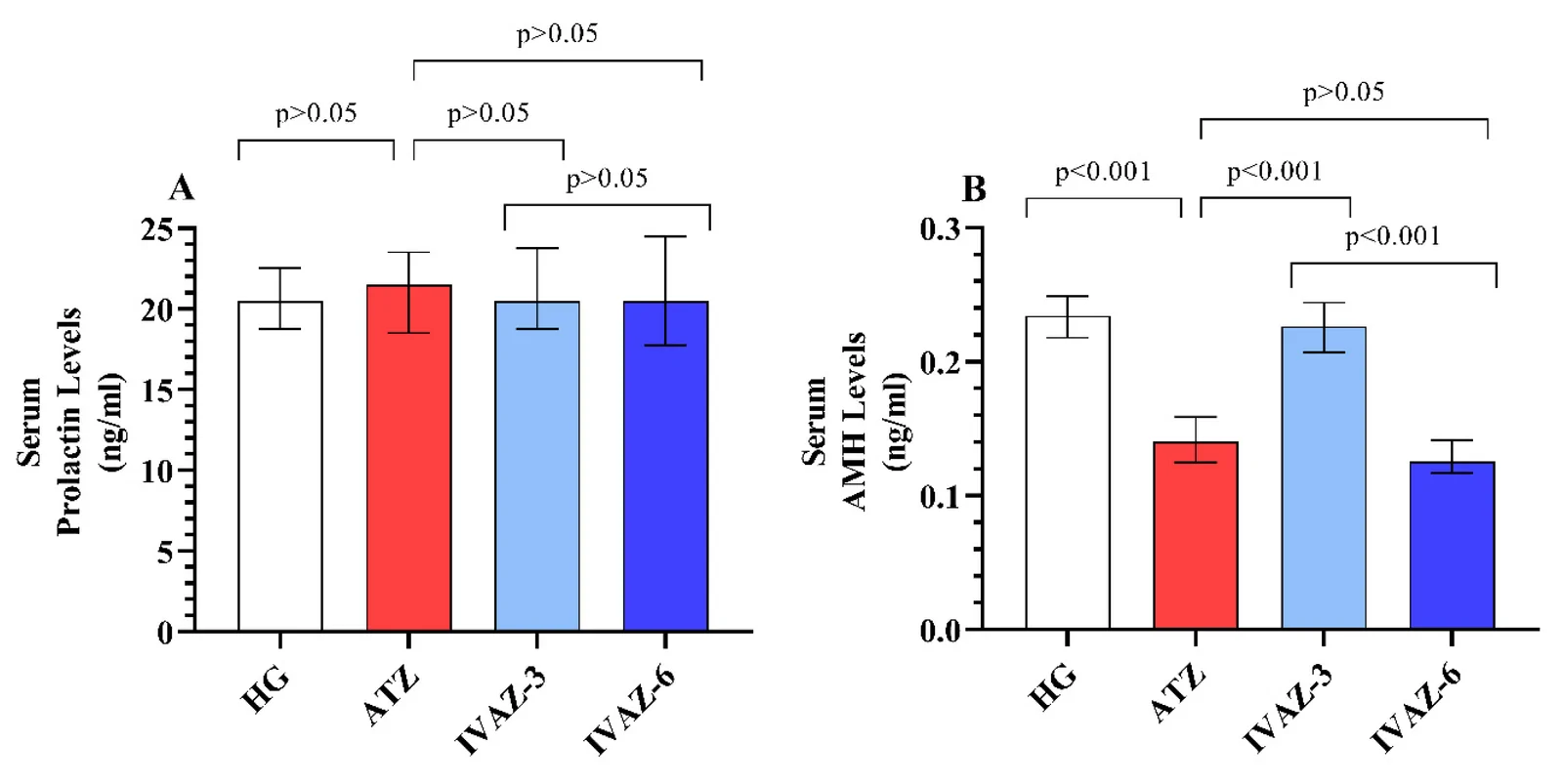
(**A**,**B**). Time-dependent effects of ivermectin treatment on serum prolactin (**A**) and anti-Müllerian hormone (**B**) levels in rats exposed to atezolizumab and treated with ivermectin for 3 or 6 days. Data are presented as means ± standard deviations (SDs), with *n* = 6 animals per group. AMH: anti-Müllerian hormone, HG: healthy group, ATZ: atezolizumab group, IVAZ-3: ivermectin + atezolizumab (3-day), IVAZ-6: ivermectin + atezolizumab (6-day). Statistical comparisons were conducted using one-way analysis of variance (ANOVA), followed by the appropriate post hoc test (Tukey test); *p* < 0.05 was considered statistically significant.

Figure 3A,B and Table 1 indicate that circulating prolactin levels remained uniform across all experimental cohorts, showing no statistically significant variations (*p* > 0.05). Conversely, serum AMH concentrations suffered a severe decline in the ATZ group compared with the HG control (*p* < 0.001). A 3-day ivermectin regimen effectively counteracted this deficit, with the IVAZ-3 group exhibiting significantly elevated AMH levels over the ATZ group (*p* < 0.001) that were statistically indistinguishable from HG values (*p* > 0.05). Extending administration to six days (IVAZ-6), however, failed to produce any meaningful recovery, as AMH levels mirrored those of the ATZ group (*p* > 0.05). Altogether, these metrics reveal that safeguarding serum AMH pools is contingent upon brief ivermectin exposure, an effect that degrades under prolonged treatment.

### 3.2. Immunohistochemical Findings

**Figure 4:**
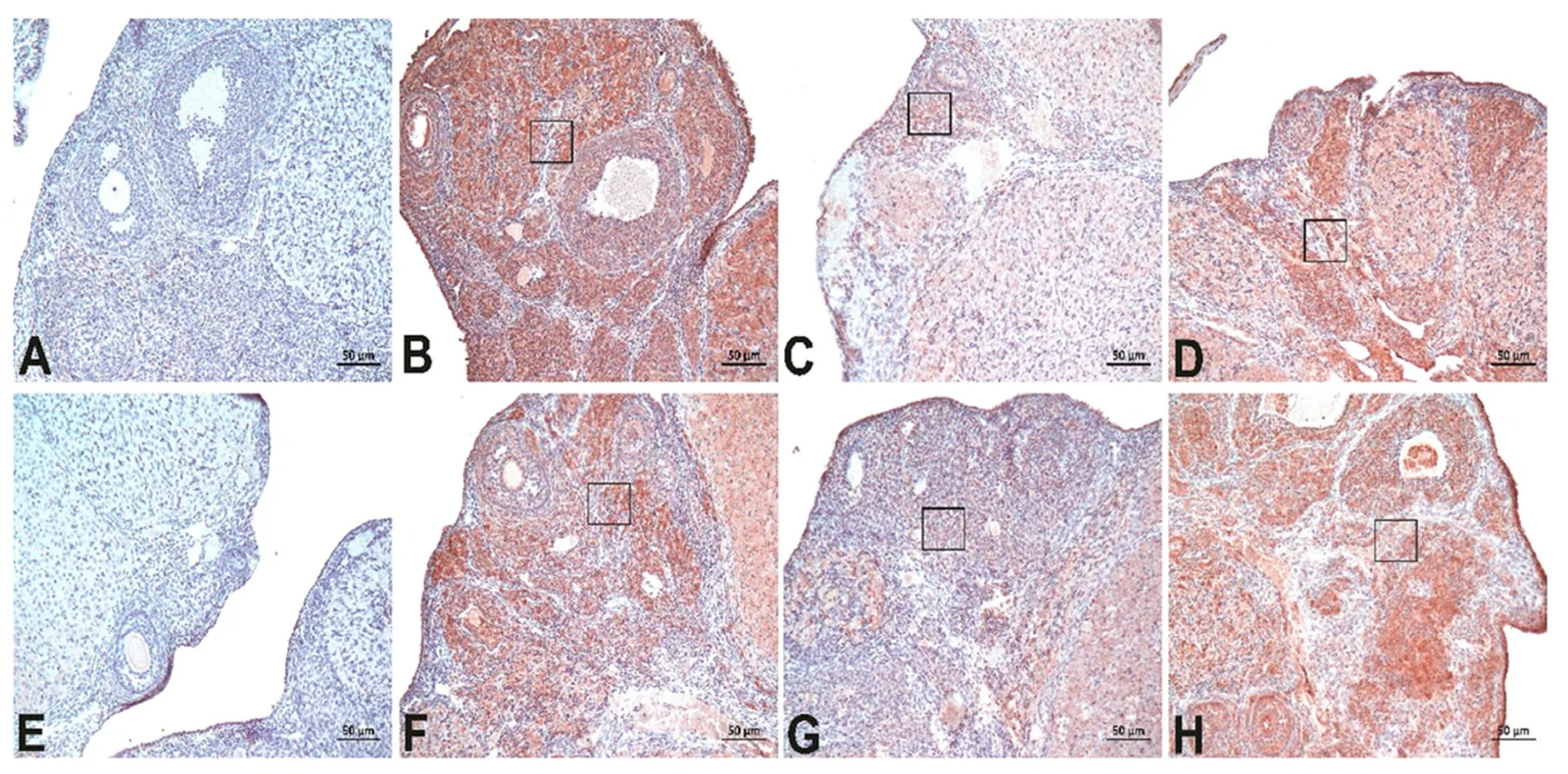
(**A**–**H**). Immunohistochemical evaluation of Caspase-3 and iNOS immunoreactivity in ovarian tissue of rats exposed to atezolizumab and treated with ivermectin for 3 or 6 days. (**A**–**D**) Caspase-3 immunoreactivity: (**A**) HG group, immunonegative; (**B**) ATZ group, severe immunopositivity; (**C**) IVAZ-3 group, mild immunoreactivity; (**D**) IVAZ-6 group, severe immunopositivity (□). (**E**–**H**) iNOS immunopositivity: (**E**) HG group, immunonegative; (**F**) ATZ group, severe immunopositivity; (**G**) IVAZ-3 group, moderate immunopositivity; (**H**) IVAZ-6 group, severe immunopositivity (□). iNOS: inducible nitric oxide synthase, HG: healthy group, ATZ: atezolizumab group, IVAZ-3: ivermectin + atezolizumab (3-day), IVAZ-6: ivermectin + atezolizumab (6-day). HG: healthy group, ATZ: atezolizumab group, IVAZ-3: ivermectin + atezolizumab (3-day), IVAZ-6: ivermectin + atezolizumab (6-day).

Caspase-3 immunopositivity was not detected in the HG group (Figure 4A), whereas intense immunopositivity was observed in the ATZ group (Figure 4B), with a significant difference between the two groups (*p* < 0.05, Table 2). In the IVAZ-3 group (Figure 4C), Caspase-3 immunopositivity decreased to a mild level and was significantly lower than that observed in the ATZ group (*p* < 0.05, Table 2). In the IVAZ-6 group (Figure 4D), Caspase-3 immunopositivity remained at a severe level, with no statistically significant difference compared with the ATZ group (*p* > 0.05, Table 2). Additionally, Caspase-3 immunopositivity was significantly lower in the IVAZ-3 group than in the IVAZ-6 group (*p* < 0.05, Table 2).

No detectable iNOS immunopositivity was observed in the HG group (Figure 4E), whereas intense iNOS immunopositivity was detected in the ATZ group (Figure 4F), showing a significant difference between the two groups (*p* < 0.05, Table 2). In the IVAZ-3 group (Figure 4G), iNOS immunopositivity decreased to a moderate level; however, the difference compared with the ATZ group was not statistically significant (*p* > 0.05, Table 2). In contrast, iNOS immunopositivity remained severe in the IVAZ-6 group (Figure 4H), and the difference from the ATZ group was not statistically significant (*p* > 0.05, Table 2). Additionally, iNOS immunopositivity differed significantly between the IVAZ-3 and IVAZ-6 groups (*p* < 0.05, Table 2).

**Figure 5:**
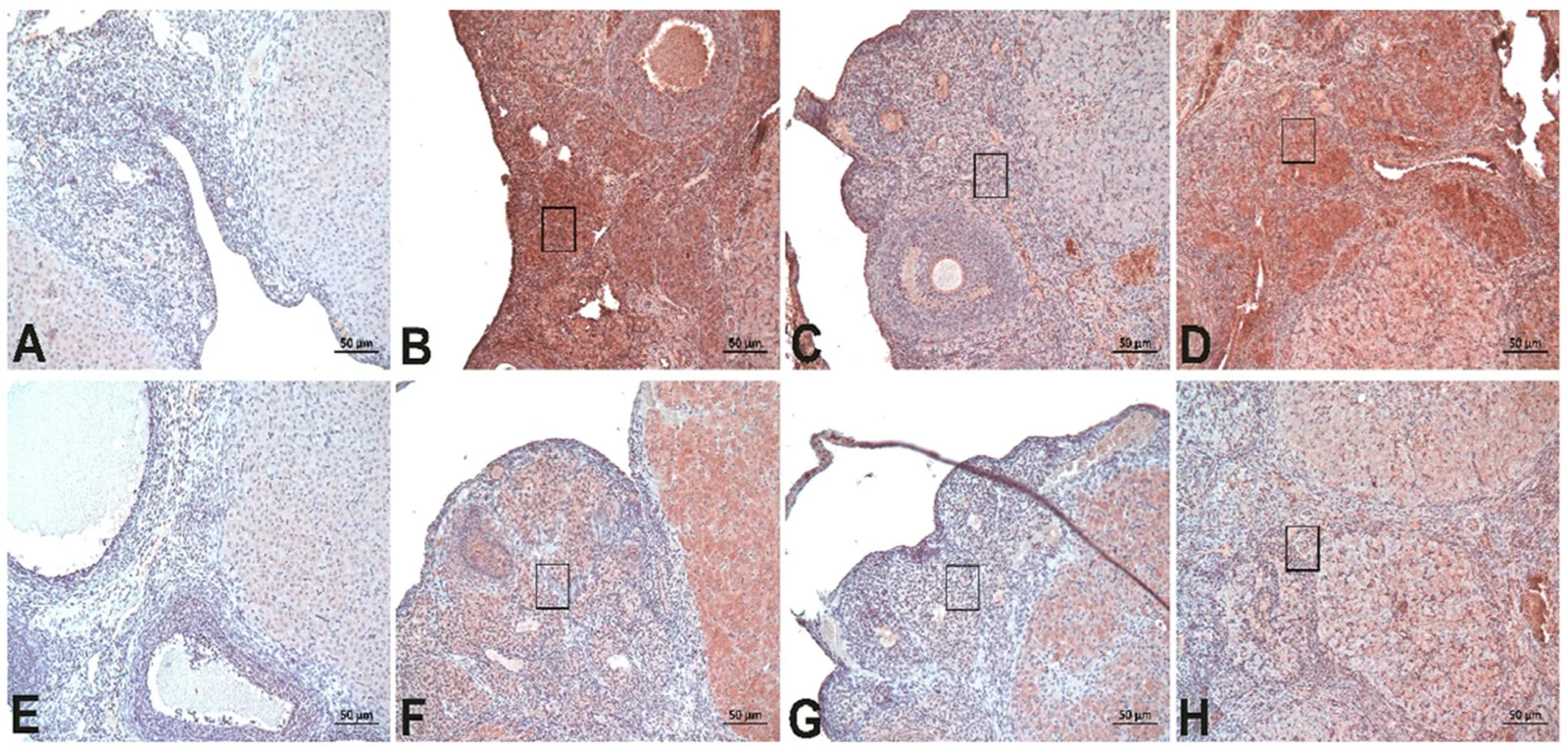
(**A**–**H**). Immunohistochemical evaluation of IL-1β and LC3B immunoreactivity in ovarian tissue of rats exposed to atezolizumab and treated with ivermectin for 3 or 6 days. (**A**–**D**) IL-1β immunoreactivity: (**A**) HG group, immunonegative; (**B**) ATZ group, very severe immunopositivity; (**C**) IVAZ-3 group, moderate immunopositivity; (**D**) IVAZ-6 group, severe immunopositivity (□). (**E**–**H**) LC3B immunoreactivity: (**E**) HG group, immunonegative; (**F**) ATZ group, severe immunopositivity; (**G**) IVAZ-3 group, moderate immunopositivity; (**H**) IVAZ-6 group, severe immunopositivity (□). IL-1β: interleukin-1 beta, LC3B: microtubule-associated protein 1 light chain 3 beta, HG: healthy group, ATZ: atezolizumab group, IVAZ-3: ivermectin + atezolizumab (3-day), IVAZ-6: ivermectin + atezolizumab (6-day).

IL-1β immunopositivity was absent in the HG group (Figure 5A), whereas very severe immunopositivity was observed in the ATZ group (Figure 5B), with a significant difference between the groups (*p* < 0.05, Table 2). In the IVAZ-3 group (Figure 5C, Table 2), IL-1β immunopositivity decreased to a moderate level and was significantly lower than that in the ATZ group (*p* < 0.05, Table 2). In the IVAZ-6 group (Figure 5D), IL-1β immunopositivity remained within the severe range; the immunopositivity scores differed significantly from those of the ATZ group (*p* < 0.05) (Figure 4, Table 2). Additionally, IL-1β immunopositivity was significantly lower in the IVAZ-3 group compared with the IVAZ-6 group (*p* < 0.05, Table 2).

LC3B immunopositivity was not detected in the HG group (Figure 5E), whereas intense immunopositivity was observed in the ATZ group (Figure 5F), with a significant difference between the two groups (*p* < 0.05). In the IVAZ-3 group (Figure 5G), LC3B immunopositivity decreased to a moderate level and was significantly lower than that in the ATZ group (*p* < 0.05). Conversely, LC3B immunopositivity remained severe in the IVAZ-6 group (Figure 5H), and no statistically significant difference was detected compared with the ATZ group (*p* > 0.05) (Figure 5, Table 2). Additionally, LC3B immunopositivity was significantly reduced in the IVAZ-3 group relative to the IVAZ-6 group (*p* < 0.05, Table 2).

### 3.3. Histopathological Findings

**Figure 6:**
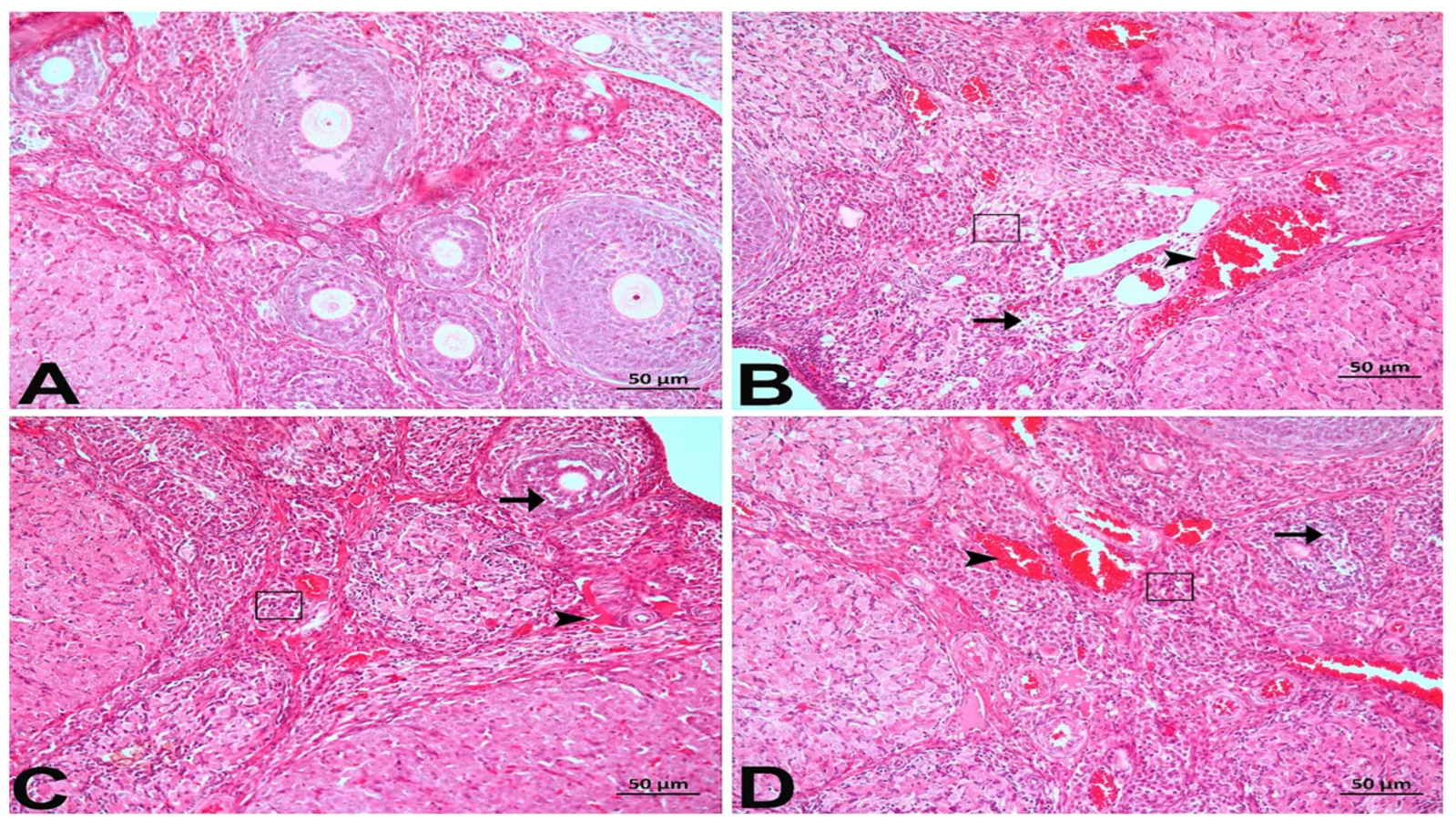
(**A**–**D**). Histopathological evaluation of ovarian tissue in rats exposed to atezolizumab and treated with ivermectin for 3 or 6 days. (**A**) Normal histological architecture. (**B**) Severe follicular (→) and parenchymal (□) degeneration with severe hyperemia (➤). (**C**) Mild follicular (→) and parenchymal (□) degeneration with mild hyperemia (➤). (**D**) Severe follicular (→) and parenchymal (□) degeneration with severe hyperemia (➤). H&E staining. HG: healthy group, ATZ: atezolizumab group, IVAZ-3: ivermectin + atezolizumab (3-day), IVAZ-6: ivermectin + atezolizumab (6-day).

Histopathological examination showed normal ovarian architecture in the HG group (Figure 6A). In contrast, the ATZ group (Figure 6B) showed severe hyperemia, follicular degeneration, and parenchymal degeneration, with significantly higher histopathological scores than the HG group (*p* < 0.05, Table 3). In the IVAZ-3 group (Figure 6C), all three pathological parameters were reduced to a mild grade, and the corresponding scores were significantly lower than those in the ATZ group (*p* < 0.05, Table 3). However, severe hyperemia, follicular degeneration, and parenchymal degeneration persisted in the IVAZ-6 group (Figure 6D), and none of the three parameters differed significantly from the ATZ group (*p* > 0.05, Table 3). Importantly, all three histopathological scores were significantly lower in the IVAZ-3 group than in the IVAZ-6 group (*p* < 0.05, Table 3).

## 4. Discussion

In the present study, the potential protective effect of ivermectin against atezolizumab-associated toxicity in ovarian tissue and reproductive function was investigated in rats using biochemical, histopathological, and immunohistochemical approaches. Atezolizumab has been associated with adverse effects affecting multiple organ systems, including the reproductive system [5,6]. Previous evidence indicates that atezolizumab may increase intracellular Ca^2+^ and ROS while simultaneously reducing cellular ATP availability [11,12,13]. Collectively, these observations raise the possibility that atezolizumab-induced reproductive toxicity may be linked to disturbances in cellular energy metabolism accompanied by an increased oxidative burden.

MDA, a prominent product of lipid peroxidation, is among the most mutagenic compounds generated during oxidative damage [35] and is frequently employed to reflect oxidative stress [36]. In the study described here, the elevated MDA concentrations detected in the atezolizumab-only group may reflect an increase in oxidative stress associated with immunotherapy exposure. This interpretation is consistent with the findings of Peruzzi et al. who reported an association between oxidative stress-related biomarkers and immune processes in patients receiving immunotherapeutic treatment. Thus, the present findings are in agreement with previously published evidence [37], further supporting a possible contribution of oxidative imbalance to atezolizumab-related ovarian injury.

In addition to MDA, tissue tGSH concentrations were assessed further to characterize the oxidative status of the ovarian tissue. GSH is a major endogenous antioxidant molecule that plays an essential role in maintaining intracellular redox homeostasis [38]. The reduction in tGSH observed in the ATZ group may indicate increased oxidative burden and enhanced utilization of GSH as part of the cellular defense response against oxidative damage [39]. A significant decline was also detected in SOD and CAT levels in the ovarian tissue, together with the reduction in tGSH. SOD and CAT represent key enzymatic components of the endogenous antioxidant defense system [24]. By regulating ROS accumulation, these enzymes contribute to limiting oxidative injury and maintaining control over inflammatory processes [40]. Accordingly, the diminished SOD and CAT observed following atezolizumab exposure may reflect impairment of the ovarian antioxidant defense capacity and may be associated with the increased oxidative stress induced by the treatment.

In the present study, ovarian tissues were additionally examined for alterations in inflammatory markers. This assessment was based on evidence that ICIs, while enhancing antitumor immunity through increased T-cell activation, may also promote the production of proinflammatory cytokines [41,42]. Previous studies have demonstrated that inhibition of the PD-1/PD-L1 signaling axis can favor macrophage polarization toward the iNOS-expressing M1 phenotype, thereby contributing to enhanced production of inflammatory mediators [43]. Excessive production of proinflammatory cytokines may exacerbate inflammatory cell infiltration and tissue injury, thereby contributing to immune-mediated toxicity in various organs [42]. In the present study, ovarian tissues from the ATZ group exhibited increased TNF-α and IL-1β levels, accompanied by pronounced IL-1β and iNOS immunopositivity. Taken together, these findings suggest that atezolizumab exposure was associated with activation of an inflammatory response within the ovarian tissue, which may contribute to the development of ovarian injury.

Increased oxidative stress can compromise mitochondrial integrity and subsequently trigger the intrinsic apoptotic pathway, with Caspase-3 serving as a key executioner of apoptotic cell death [44]. Activation of this pathway may contribute to granulosa cell depletion, follicular atresia, and deterioration of ovarian reserve [45]. However, our literature search revealed no studies specifically addressing the effects of atezolizumab on Caspase-3 immunoreactivity in ovarian tissue. Nevertheless, evidence from a liver injury model has demonstrated that ICI exposure can enhance Caspase-3 expression and promote apoptotic cell death [46]. Oxidative stress and excessive ROS accumulation may also activate autophagy, an adaptive cellular process involved in maintaining intracellular homeostasis in response to stress. Atezolizumab has been reported to induce autophagy in osteosarcoma cells, accompanied by increased LC3B expression [33]. In the present study, the pronounced Caspase-3 and LC3B immunopositivity observed in the ATZ group suggests that atezolizumab exposure may simultaneously engage apoptotic and autophagic pathways in ovarian tissue, potentially as a consequence of oxidative stress.

Serum prolactin and AMH concentrations were also examined in relation to the potential reproductive effects of atezolizumab. Prolactin has been described not as a primary immunoregulatory mediator of the immune system, but rather as a hormone with anabolic and stress-modulating functions in a variety of cell types, including immune cells [47]. The absence of a significant alteration in serum prolactin following atezolizumab exposure may suggest that prolactin is not consistently sensitive to inflammatory changes and that its response may vary according to the tissue involved and the intensity of the inflammatory process. In addition to prolactin, AMH concentrations are widely used to assess reproductive function and ovarian reserve. Therefore, serum AMH was evaluated alongside prolactin in the present study. AMH is considered an important indicator of ovarian reserve and reproductive potential [48], and previous studies have suggested an inverse relationship between AMH concentrations and inflammatory activity [49]. Accordingly, the reduction in AMH levels observed following atezolizumab exposure may be related to depletion of the follicular reserve resulting from enhanced T-cell activation associated with immune checkpoint inhibition and the accompanying inflammatory injury in ovarian tissue [50]. In this context, the lower AMH concentrations detected in the ATZ group may reflect impaired ovarian reserve in association with the inflammatory response induced by atezolizumab.

The biochemical and immunohistochemical evidence derived from this work was consistent with the histopathological observations. In the ATZ group, prominent hyperemia, follicular degeneration, and parenchymal degeneration were evident on histological examination. These structural alterations further support the occurrence of ovarian tissue injury associated with ICI exposure. Supporting this interpretation, Winship et al. reported that ICI treatment may increase immune cell infiltration within ovarian tissue, accompanied by depletion of the follicular reserve, impaired oocyte maturation, and reduced ovulatory capacity [10].

In the current study, which investigated the potential protective role of ivermectin against atezolizumab-associated ovarian toxicity, ivermectin was considered to have the capacity to counteract intracellular Ca^2+^ accumulation, oxidative stress, and cellular injury, effects that have also been demonstrated in experimental models [51]. Ivermectin can additionally act as a positive allosteric modulator of ATP-gated P2 × 4 receptors, thereby increasing the probability of ion channel opening and enhancing ATP-mediated cellular responses [52,53]. Conversely, ivermectin has also been reported to induce cell death through mitochondrial pathways and to cause a time-dependent decline in intracellular ATP levels [26]. In the IVAZ-3 group, the significantly lower MDA concentration together with the higher antioxidant parameters compared with the ATZ group suggests that ivermectin, at the dose and treatment duration used in this protocol, effectively attenuated oxidative stress. This interpretation is supported by previous reports showing that ivermectin can mitigate infection-associated oxidative stress, reduce MDA accumulation, and preserve antioxidant defenses, including SOD, CAT, and GSH [54]. However, in the IVAZ-6 group, MDA and antioxidant parameters approached the levels observed in the ATZ group. This pattern suggests that the beneficial effect of ivermectin may be influenced by both treatment duration and cumulative exposure. The finding is also compatible with previous evidence indicating that prolonged or excessive ivermectin exposure may diminish its favorable effects on antioxidant defense and, under certain conditions, contribute to oxidative stress [55]. Collectively, the marked improvement in oxidative stress parameters observed in the IVAZ-3 group suggests that ivermectin may attenuate atezolizumab-induced tissue injury and preserve ovarian cellular homeostasis. Given that maintaining redox equilibrium is indispensable for follicular integrity and physiological function [22], these findings highlight the novel therapeutic potential of ivermectin as an adjuvant protective agent against atezolizumab-associated ovarian toxicity.

Ivermectin has been reported to exert anti-inflammatory activity through suppression of nuclear factor kappa B (NF-κB) and signal transducer and activator of transcription 3 (STAT3) signaling pathways, consequently reducing the production of proinflammatory cytokines [53]. The suppression of the increase in proinflammatory cytokine levels observed in the IVAZ-3 group, together with the reduced IL-1β and iNOS immunopositivity, supports the anti-inflammatory effect of ivermectin, potentially through inhibition of these signaling pathways. Consistent with this interpretation, Zhang et al. highlighted the anti-inflammatory potential of ivermectin and demonstrated that it suppressed iNOS expression in lipopolysaccharide-stimulated macrophages [24]. Conversely, the pronounced attenuation of this protective effect in the high-dose ivermectin group suggests that its anti-inflammatory efficacy may be dose-dependent and less pronounced at higher exposure levels, potentially limiting its capacity to preserve tissue integrity. From a biological perspective, the attenuation of inflammatory signaling and iNOS expression may be relevant to the preservation of ovarian tissue integrity, as excessive inflammatory activation can contribute to cellular injury and functional impairment [24,37]. These findings further support the potential of ivermectin as a protective agent against atezolizumab-associated ovarian toxicity, although the duration-dependent nature of the observed effect highlights the need for further studies to define an appropriate exposure regimen and to clarify its therapeutic relevance.

Despite atezolizumab exposure, the 3-day ivermectin regimen was associated with a marked reduction in Caspase-3 immunopositivity in ovarian tissue. This observation is in line with findings from a 7,12-dimethylbenz(a)anthracene (DMBA)-induced model of ovarian toxicity, where ivermectin was shown to counteract the increase in Caspase-3 expression and mitigate ovarian tissue injury [24]. A similar pattern was observed for LC3B, with weaker immunopositivity detected following the 3-day ivermectin protocol. To our knowledge, the influence of ivermectin on LC3B immunoreactivity in ovarian tissue has not previously been characterized. Although ivermectin has been reported to enhance LC3B expression through autophagy activation in other cellular systems [56], the discrepancy with our findings may be attributable to differences in experimental conditions, target tissue, and treatment exposure. The concurrent reduction in Caspase-3 and LC3B immunoreactivity may indicate attenuation of cell death- and stress-related responses, suggesting that ivermectin may contribute to the preservation of ovarian cellular integrity.

The histopathological findings provided further evidence for the beneficial effect of the 3-day ivermectin protocol. Marked regression of hyperemia, follicular degeneration, and parenchymal degeneration in the IVAZ-3 group agrees with previous observations that ivermectin can attenuate histological alterations in DMBA-induced ovarian toxicity [22]. In contrast, these morphological improvements were not evident to the same extent after the 6-day regimen. The histopathological findings were consistent with the biochemical and immunohistochemical results, suggesting that the tissue-protective effect of ivermectin may depend on treatment duration. The preservation of follicular and parenchymal architecture in the IVAZ-3 group may reflect reduced cellular injury and better maintenance of ovarian tissue integrity.

Serum prolactin concentrations remained unchanged following ivermectin treatment relative to the ATZ group. This observation is compatible with the view that prolactin may have limited sensitivity as an indicator of inflammatory activity, with its biological response potentially varying according to the affected tissue and the magnitude of the inflammatory process [47]. To our knowledge, the relationship between ivermectin exposure and serum AMH concentrations has not been directly characterized [57]. In the present study, the higher AMH concentrations observed in the IVAZ-3 group compared with the ATZ group, with values approaching those of the HG group, may therefore provide preliminary evidence of a favorable effect of the 3-day ivermectin regimen on ovarian reserve. Given that AMH is widely used as an indicator of ovarian reserve [48], the preservation of serum AMH in the IVAZ-3 group may indicate that the observed tissue-level protective effects of ivermectin were accompanied by preservation of an important functional marker of ovarian reserve. In contrast, the absence of a comparable improvement after the 6-day regimen suggests that this potential benefit may also be influenced by treatment duration. Although these findings do not establish a direct effect of ivermectin on ovarian reserve, they provide a biologically relevant basis for further investigation of ivermectin as a potential protective strategy against treatment-associated ovarian dysfunction.

Overall, the present findings suggest that ivermectin may have potential as a protective strategy against atezolizumab-associated ovarian toxicity. The improvement in oxidative stress and inflammatory parameters, together with the preservation of AMH levels in the IVAZ-3 group, is particularly relevant because ovarian reserve and reproductive function are important aspects of long-term health in women receiving anticancer therapy. The greater protective effect observed with the shorter treatment duration further suggests that the timing of ivermectin exposure may be an important determinant of its efficacy. These findings provide a potential basis for further investigation of ivermectin as a pharmacological approach to preserve ovarian function during immune checkpoint inhibitor therapy. Future studies should determine whether this protective effect can be achieved without compromising the antitumor activity of atezolizumab and should further clarify the optimal timing and duration of ivermectin treatment.

Limitations: Certain limitations of this investigation should be considered. First, the experimental design included only two ivermectin treatment durations and did not incorporate a comprehensive dose–response assessment across different ivermectin doses. Second, although ovarian injury was characterized using biochemical, histopathological, and immunohistochemical endpoints, reproductive performance itself was not directly evaluated. Furthermore, the antitumor efficacy of atezolizumab was not assessed. Consequently, the present findings do not allow us to determine whether the potential ovarian protective effects of ivermectin interfere with the therapeutic or immunological activity of atezolizumab. In addition, estrous cycle stages were not monitored or synchronized, and therefore, potential cycle-related variations in ovarian biochemical, histopathological, and hormonal parameters cannot be completely excluded. Finally, FSH levels were not assessed, which could have provided additional information regarding ovarian endocrine function.

## 5. Conclusions

The present study demonstrated that atezolizumab exposure was associated with increased oxidative stress, inflammatory activity, apoptotic and autophagic responses, and histopathological alterations in rat ovarian tissue, accompanied by a reduction in serum AMH concentrations. Ivermectin substantially attenuated these alterations, particularly under the 3-day treatment protocol. This protective pattern was supported by the biochemical and immunohistochemical findings, the marked regression of ovarian hyperemia and follicular and parenchymal degeneration, and the preservation of serum AMH concentrations in the IVAZ-3 group. In contrast, the protective response was not pronounced following the 6-day ivermectin regimen, suggesting that the ovarian effects of ivermectin may be influenced not only by the exposure level but also by treatment duration. These findings raise the possibility that the antioxidant and anti-inflammatory properties of ivermectin may be more evident under shorter-term, appropriately dosed conditions, whereas prolonged exposure may attenuate these beneficial effects. Although the present findings provide preliminary experimental evidence supporting a potential protective role for ivermectin against atezolizumab-associated ovarian toxicity, the underlying mechanisms and clinical relevance remain to be established. Overall, ivermectin may represent a potential protective agent against atezolizumab-induced ovarian injury, particularly when administered for a shorter duration; however, further comprehensive experimental studies and clinical investigations are warranted to determine whether this protective effect translates into meaningful preservation of reproductive function.

## Figures and Tables

**Table 1:** Biochemical parameters in ovarian tissue and serum of rats exposed to atezolizumab and treated with ivermectin for 3 or 6 days.

Parameters		Experimental Groups (*n* = 6 Per Group)			
		HG	ATZ	IVAZ-3	IVAZ-6
		Mean ± Standard Deviation			
Ovarian tissues	MDA (nmol/mg protein)	6.24 ± 0.20	9.44 ± 0.17 *	5.44 ± 0.26 *^,^**	9.55 ± 0.35 *^,^#
	tGSH (ng/mg protein)	8.42 ± 0.23	5.36 ± 0.17 *	8.02 ± 0.26 *^,^**	5.14 ± 0.33 *^,^#
	SOD (ng/mg protein)	8.51 ± 0.27	4.84 ± 0.26 *	7.69 ± 0.29 *^,^**	4.73 ± 0.56 *
	CAT (ng/mg protein)	5.81 ± 0.11	3.12 ± 0.19 *	5.20 ± 0.16 *^,^**	2.91 ± 0.41 *^,^#
	TNF-α (ng/mg protein)	3.39 ± 0.19	5.78 ± 0.29 *	3.70 ± 0.14 **	5.89 ± 0.36 *^,^#
	IL-1β (pg/mg protein)	2.40 ± 0.26	4.47 ± 0.19 *	2.79 ± 0.43 **	4.36 ± 0.20 *^,^#
Serum	Prolactin (ng/mL)	20.67 ± 2.17	21.17 ± 2.99	21.17 ± 2.93	21.00 ± 2.74
	AMH (ng/mL)	0.23 ± 0.02	0.14 ± 0.02 *	0.23 ± 0.02 **	0.13 ± 0.02 *^,^#

*: *p* < 0.05 vs. HG, **: *p* < 0.05 vs. ATZ, #: *p* < 0.05 vs. IVAZ-3, MDA: Malondialdehyde, tGSH: total glutathione, SOD: superoxide dismutase, CAT: catalase, TNF-α: tumor necrosis factor-alpha, IL-1β: interleukin-1 beta, AMH: anti-Müllerian hormone, HG: healthy group, ATZ: atezolizumab group, IVAZ-3: ivermectin + atezolizumab (3-day), IVAZ-6: ivermectin + atezolizumab (6-day). Statistical comparisons were conducted using one-way analysis of variance (ANOVA), followed by the appropriate post hoc test (Tukey or Games–Howell tests); *p* < 0.05 was considered statistically significant.

**Table 2:** Semi-quantitative immunohistochemical analysis of Caspase-3, iNOS, IL-1β, and LC3B immunoreactivity in ovarian tissue of rats exposed to atezolizumab and treated with ivermectin for 3 or 6 days.

Parameters	Experimental Groups (*n* = 6 Per Group)			
	HG	ATZ	IVAZ-3	IVAZ-6
	Median (25th–75th Percentile)			
Caspase 3	0 (0–0.25)	3 (3–3) *	1 (1–2) *^,^**	3 (2.75–3) *^,^#
iNOS	0 (0–0.25)	3 (2.75–3) *	2 (2–3) *	3 (3–3) *^,^#
IL-1β	0 (0–1)	4 (3.75–4) *	2 (2–2) *^,^**	3 (3–3.25) *^,^**^,^#
LC3B	0 (0–1)	3 (3–3.25) *	2 (2–3) *^,^**	3 (3–3) *^,^#

Immunopositivity: 0: absent, 1: mild, 2: moderate, 3: severe, 4: very severe. *: *p* < 0.05 vs. HG, **: *p* < 0.05 vs. ATZ, #: *p* < 0.05 vs. IVAZ-3. iNOS: Inducible nitric oxide synthase, IL-1β: Interleukin-1 beta, LC3B: Microtubule-associated protein 1A/1B-light chain 3B, HG: healthy group, ATZ: atezolizumab group, IVAZ-3: ivermectin + atezolizumab (3-day), IVAZ-6: ivermectin + atezolizumab (6-day). Statistical analysis: Kruskal–Wallis test followed by pairwise Mann–Whitney U tests between groups; *p* < 0.05 was considered significant.

**Table 3:** Histopathological damage scores in ovarian tissue of rats exposed to atezolizumab and treated with ivermectin for 3 or 6 days.

Parameters	Experimental Groups (*n* = 6 Per Group)			
	HG	ATZ	IVAZ-3	IVAZ-6
	Median (25th–75th Percentile)			
Hyperemia	0 (0–0)	3 (2.75–3) *	1 (1–2) *^,^**	3 (2–3) *^,^#
Follicular degeneration	0 (0–0)	3 (2–3) *	1 (1–1) *^,^**	3 (2–3) *^,^#
Parenchymal degeneration	0 (0–0)	3 (2.75–3) *	1 (0.75–1) *^,^**	2.5 (2–3) *^,^#

Histopathological damage scoring: 0: absent, 1: mild, 2: moderate, 3: severe. *: *p* < 0.05 vs. HG, **: *p* < 0.05 vs. ATZ, #: *p* < 0.05 vs. IVAZ-3. HG: healthy group, ATZ: atezolizumab group, IVAZ-3: ivermectin + atezolizumab (3-day), IVAZ-6: ivermectin + atezolizumab (6-day). Statistical analysis: Kruskal–Wallis test followed by pairwise Mann–Whitney U tests between groups; *p* < 0.05 was considered significant.

## Data Availability

The original contributions presented in this study are included in the article. Further inquiries can be directed to the corresponding author.
